# Transcriptome-Wide Association Supplements Genome-Wide Association in *Zea mays*

**DOI:** 10.1534/g3.119.400549

**Published:** 2019-07-23

**Authors:** Karl A. G. Kremling, Christine H. Diepenbrock, Michael A. Gore, Edward S. Buckler, Nonoy B. Bandillo

**Affiliations:** * Institute for Genomic Diversity; † Plant Breeding and Genetics Section, School of Integrative Plant Science, Cornell University, Ithaca, NY 14853, and; ‡ United States Department of Agriculture-Agricultural Research Service, Robert W. Holley Center for Agriculture and Health, Ithaca, NY 14853

**Keywords:** endophenotypes, Fisher’s combined test, genome-wide association studies, natural variation, transcriptome-wide association studies, variance partitioning

## Abstract

Modern improvement of complex traits in agricultural species relies on successful associations of heritable molecular variation with observable phenotypes. Historically, this pursuit has primarily been based on easily measurable genetic markers. The recent advent of new technologies allows assaying and quantifying biological intermediates (hereafter endophenotypes) which are now readily measurable at a large scale across diverse individuals. The usefulness of endophenotypes for delineating the regulatory landscape of the genome and genetic dissection of complex trait variation remains underexplored in plants. The work presented here illustrated the utility of a large-scale (299-genotype and seven-tissue) gene expression resource to dissect traits across multiple levels of biological organization. Using single-tissue- and multi-tissue-based transcriptome-wide association studies (TWAS), we revealed that about half of the functional variation acts through altered transcript abundance for maize kernel traits, including 30 grain carotenoid abundance traits, 20 grain tocochromanol abundance traits, and 22 field-measured agronomic traits. Comparing the efficacy of TWAS with genome-wide association studies (GWAS) and an ensemble approach that combines both GWAS and TWAS, we demonstrated that results of TWAS in combination with GWAS increase the power to detect known genes and aid in prioritizing likely causal genes. Using a variance partitioning approach in the largely independent maize Nested Association Mapping (NAM) population, we also showed that the most strongly associated genes identified by combining GWAS and TWAS explain more heritable variance for a majority of traits than the heritability captured by the random genes and the genes identified by GWAS or TWAS alone. This not only improves the ability to link genes to phenotypes, but also highlights the phenotypic consequences of regulatory variation in plants.

Discovery of variation that underlies quantitative traits remains central to the genetic improvement of agricultural species. Functional variation can alter coding sequence or act to regulate an intermediate phenotype. Regulating the abundance of phenotypic intermediates, such as mRNA expression or protein level, provides a more spatially and temporally subtle target for selection than coding sequence changes, which are more likely to be pleiotropic and therefore maladaptive (Mayr 1970). Thus, regulatory variation is the frequent target of both natural and artificial selection that shapes genomes across life, including domesticated plants (Carroll 2008; Hufford *et al.* 2012; Mayr 1970). It is likely that about half of functional variation is regulatory (Albert and Kruglyak 2015; Gusev *et al.* 2014; Rodgers-Melnick *et al.* 2016; Welter *et al.* 2014). It should also be noted that regulation can take place at any biological level of organization from the epigenetic state (Law and Jacobsen 2010), to gene expression (Albert and Kruglyak 2015; Fu *et al.* 2013; GTEx Consortium 2015), to ribosome occupancy (Juntawong *et al.* 2014), to metabolites (Riedelsheimer *et al.* 2012), to protein abundance (Battle *et al.* 2015; Chick *et al.* 2016), furnishing multiple levels at which intermediate and terminal phenotypes can be associated.

In standard genetic mapping approaches, like association or linkage mapping, associations between genetic markers and terminal phenotypes of interest are tested for significance (black arrow, Figure 1). However, multiple levels of biological organization exist between the DNA sequence and the terminal observed phenotypic outcomes, enabling trait dissection to be conducted between intermediate levels of biological organization (hereafter endophenotypes, designated by an orange and red arrow in Figure 1). Associating endophenotypes with terminal phenotypes predates the use of molecular genetic markers for mapping. The use of linked observable traits and isozyme migration patterns are examples of tying markers from biological intermediates to terminal phenotypes of interest. Similarly, just as relationships between individuals can be calculated from molecular genetic markers (Flint-Garcia *et al.* 2005), endophenotypic similarity from isozyme markers can also be used to quantify relatedness (Dubreuil and Charcosset 1998). These same principles have recently been extended to phenotypic prediction guided by metabolites (Riedelsheimer *et al.* 2012) or by expression dysregulation (Kremling *et al.* 2018). However, the use of molecular intermediates, which are now readily measurable at large scale across diverse individuals, remains underexplored in plants for the inverse task of causal inference.

**Figure 1:**
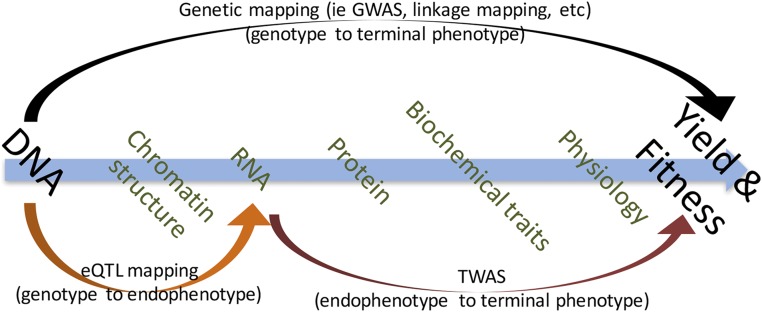
Levels of biological organization between the ultimate cause of genetics and the terminal phenotypic outcomes can be exploited individually to improve power and inference of biological mechanism. Genotype can be linked to endophenotype as in eQTL or protein QTL (pQTL), or endophenotype can be linked to terminal phenotype by methods like TWAS.

Associating endophenotypes with terminal phenotypes has multiple distinct advantages. First, while genetic mapping is dominated by the covariance structure of neighboring SNPs and complex haplotypes, endophenotypes provide orthogonal information that often permits inference regarding biological mechanism, which may not be possible from genetic variants alone. Second, genetic mapping often points to intergenic (Wallace *et al.* 2014) regulatory variants that are not within the coding sequence of the gene that alters the phenotype (Albert and Kruglyak 2015). Therefore, an association signal cannot directly be tied to a corresponding gene and may even be in the body of a second unrelated gene (Tishkoff *et al.* 2007) or in the case of synthetic association, between multiple true causal variants affecting different genes (Dickson *et al.* 2010). Association tests with intermediate expression phenotypes do not suffer from these limitations. Third, the abundance of endophenotypes is largely independent of linkage disequilibrium (LD), unlike in the case of genetic markers. In other words, even multiple genes that are perfectly linked, and thus not independently observable in separate individuals, can be prioritized for association with a trait because their expression patterns are independent. This is of greatest utility in species where linkage disequilibrium is extensive or where making high-resolution mapping populations is not feasible.

Intermediate phenotypes, such as expression, can also integrate the signal from changes in multiple components of a network, which may not be individually detectable either because their effects are small or changes to the peripheral network components occur at low frequencies. Similarly, intermediate phenotypes can integrate a phenotypic signal from underlying genetic variants for which low frequencies preclude direct detection. The most deleterious of variants are expected to segregate at the lowest frequencies (Gibson 2012; Henn *et al.* 2015) and, thus, escape detection by mapping without prohibitively large sample sizes. However, rare deleterious variants can be expected to drive common maladaptive patterns in intermediate phenotypes that are thus more easily detected through endophenotype association tests like transcriptome-wide association studies (TWAS) (Hirsch *et al.* 2014; Pasaniuc and Price 2017). Methods for integrating expression association tests with GWAS have also been used extensively in the human context as shown by Gusev *et al.* (2016) and Mancuso *et al.* (2017). However, those methods rely on summary statistics, LD scores, and expression imputation and are computationally more intensive than the more accessible Fisher’s combined test whose utility and improved power over TWAS or GWAS alone we have shown here for the first time and recommend for other researchers in model contexts.

Here, we illustrate the power of using gene expression endophenotypes measured in a large 299-individual, seven-tissue gene expression resource (Kremling *et al.* 2018) collected from the Goodman maize diversity panel (Flint-Garcia *et al.* 2005). Expression levels are correlated with terminal phenotypes in TWAS (Hirsch *et al.* 2014; Pasaniuc and Price 2017) and then combined with genotype-based associations from GWAS. The method is demonstrated here in a maize inbred diversity panel (Flint-Garcia *et al.* 2005), which has been widely used to dissect the architecture of dozens of traits of varying complexity (Harjes *et al.* 2008; Lipka *et al.* 2013; Owens *et al.* 2014; Wisser *et al.* 2011).

Related work in maize that relies on associating expression differences directly with phenotype using a Bayesian method, called expression read depth GWAS (eRD-GWAS), has been published recently (Lin *et al.* 2017). This work used 369 maize samples from which shoot apex RNA was collected. Beyond the difference in frequentist *vs.* Bayesian approaches, our study also exploits expression measurements from seven tissues in a multiple-regression-based TWAS and integrates the signal from TWAS and GWAS into a more powerful combined test which can be readily visualized as a Manhattan plot. We also compare the power of each model based on the ability to detect known genes, and the capacity to explain variance in a separate population, which differs from the approach of the previous study (Lin *et al.* 2017). To make this comparison we use the maize NAM population (Yu *et al.* 2008), which has the advantage of being largely independent of the diversity panel (Flint-Garcia *et al.* 2005) in which detection was performed.

We assess the efficacy of TWAS by quantifying the capacity to identify previously identified genes, and by the fraction of phenotypic variance explained (Gusev *et al.* 2014; Rodgers-Melnick *et al.* 2016) by the most strongly associated genes, and compared the TWAS results with GWAS and an ensemble approach combining both TWAS and GWAS. We illustrate that the results of TWAS are a valuable supplement to GWAS mapping that aids in prioritizing likely causal genes when both methods are used in a combined test.

## Materials and Methods

### Genotypic data

Genotypes for the Goodman diversity panel (Flint-Garcia *et al.* 2005) used in the genome-wide association studies were from the unimputed maize HMP 3.2.1 called against the B73 reference genome (Bukowski *et al.* 2018). Variants segregating above 5% minor allele frequency (MAF) in the union of all lines were considered for mapping. Variance component estimation in the maize NAM population (Yu *et al.* 2008) was performed using imputed HMP 3.2.1 variants [filename: NAM_HM321_KNN.hmp.txt.gz].

### Phenotypic data

For mapping in the Goodman diversity panel, kernel carotenoid BLUPs from 30 traits were from Owens *et al.* (2014) and the 20 kernel tocochromanol traits BLUPs were from Lipka *et al.* (2013) after additional outliers were removed. The 22 field-based agronomic trait BLUPs were those calculated by Hung *et al.* (2012). Phenotypes used in variance partitioning with the maize NAM population were from Diepenbrock *et al.* (2017) for the tocochromanol traits. Agronomic trait BLUPs were previously calculated by Hung *et al.* (2012).

### Expression data

Expression quantifications were those created from seven diverse tissues in maize by aligning 3′ mRNAseq reads against the AGPv3.29 maize genome as described by Kremling *et al.* (2018).

### Genome-wide association study

Genome-wide association tests were conducted in the maize Goodman diversity panel (Flint-Garcia *et al.* 2005) using a mixed linear model as implemented in FastLMM (Lippert *et al.* 2011) accounting for kinship and a naive general linear model fit using MatrixEQTL (Shabalin 2012) as implemented in TASSEL (Bradbury *et al.* 2007).

### Transcriptome-wide association study

Transcriptome-wide association tests were conducted in the maize Goodman diversity panel (Flint-Garcia *et al.* 2005) for genes that were expressed in at least half of individuals represented in a specific tissue. A linear model was fit individually for each phenotype*expressed gene combination in which the explanatory variable is the expression value of a gene across individuals. TWAS was attempted both without covariates and with five genetic principal coordinates (calculated from maize HMP3.2.1 used in (Kremling *et al.* 2018) and 25 probabilistic estimation of expression residuals (PEER) hidden factors (calculated separately for each tissue) as calculated in (Kremling *et al.* 2018). Multi-tissue TWAS was also performed. First a model was fit once per trait using the five principal coordinates described above. This model was then compared by analysis of variance (ANOVA) to a model for each gene containing terms for each tissue and the principal coordinates. The p-value resulting from this ANOVA was used to determine whether the multi-tissue model is significantly better than the covariate-only model. This p-value was also used as the p-value in the second of the Fisher’s combined tests below.

### Fisher’s combined tests of TWAS and GWAS

The GWAS p-value (mixed linear model with kinship as a random effect) of each SNP in the top 10% of most associated SNPs was assigned to nearest gene and then combined with the TWAS p-value (linear model with multi-dimensional scaling (MDS) principal coordinates + PEERs) for that same gene using Fisher’s combined test as implemented in the sumlog method in the *metap* package (Dewey 2017) in R. TWAS p-values for genes which were not tested in TWAS (*i.e.*, their expression was not observed in at least half of individuals) were set to *P* = 1 prior to combining with GWAS p-values. Fisher’s combined tests were performed in the same way when including the multi-tissue TWAS results instead of the kernel-only results.

### Variance partitioning

Using the k-Nearest Neighbors (KNN) imputed Nested Association Mapping population HMP3.2.1 genotypes described above, kinship matrices were calculated based on the top ten genes identified by each of the TWAS, GWAS, and combined models described in the Goodman diversity panel (Flint-Garcia *et al.* 2005). To independently assess the accuracy of detected genes, the phenotypic variance explained by each kinship matrix was calculated in the Nested Association Mapping population, within each family and across all the NAM families. For TWAS, the top 10 genes were taken and all SNPs within a 0.5 Mb radius of the start and end of the gene (maize annotation AGPv3.29) were used to calculate a single kinship matrix per trait using the Variance Component Annotation Pipeline in TASSEL (Bradbury *et al.* 2007). The REML solver in LDAK (Speed *et al.* 2017) was used to calculate the variance explained by the single kinship matrix. For GWAS, the SNPs were ordered based on significance and assigned to their nearest gene. The top ten unique genes from this list were taken to calculate kinship matrices using the same 0.5 Mb radius around the gene. To avoid picking multiple genes and redundant variants from the same peak based on the GWAS results, the top most associated gene was used within a peak and all other genes within the 0.5 Mb radius were excluded from selection as top genes.

### Overlap with known kernel metabolite genes

Fourteen known tocochromanol biosynthetic genes identified in NAM (Diepenbrock *et al.* 2017) and 58 *a priori* candidate genes relevant to the biosynthesis and retention of carotenoids (Owens *et al.* 2014) were used as positive controls to test the capacity of our GWAS, TWAS, and combined methods to re-detect known genes. In order to avoid comparison of p-value thresholds across methods, positive detections were counted if a gene was detected among the top 1% of genes associated with a trait.

### Data availability

All data are held in public repository. The SNP data for the Goodman diversity panel (Flint-Garcia *et al.* 2005) used in the genome-wide association studies were from the unimputed maize HMP 3.2.1 called against the B73 reference genome (Bukowski *et al.* 2018). The imputed HMP 3.2.1 variants [filename: NAM_HM321_KNN.hmp.txt.gz] for the maize NAM population (Yu *et al.* 2008) was used for the variance component estimation. Expression quantifications were those created from seven diverse tissues in maize by aligning 3′ mRNAseq reads against the AGPv3.29 maize genome as described by Kremling *et al.* (2018). Kernel carotenoid BLUPs from 30 traits were from Owens *et al.* (2014) and the 20 kernel tocochromanol traits BLUPs were from Lipka *et al.* (2013) after additional outliers were removed. The 22 field-based agronomic trait BLUPs were those calculated by Hung *et al.* (2012). Phenotypes used in variance partitioning with the maize NAM population were from Diepenbrock *et al.* (2017) for the tocochromanol traits. Agronomic trait BLUPs were previously calculated by Hung *et al.* (2012). Supplemental material available at Figshare: https://figshare.com/s/ef57544b4d09d5c55131.

## Results

To test the utility of expression data in dissecting quantitative traits in maize, we performed single-tissue-based and multi-tissue-based TWAS (Pasaniuc and Price. 2017) and compared these results with GWAS results, and an ensemble approach combining GWAS and TWAS results using the Fisher’s combined test. In TWAS, expression levels across seven tissues from a maize diversity panel (Flint-Garcia *et al.* 2005) were used individually and together in a multiple regression as independent variables and correlated with previously measured phenotypes for maize kernel traits, including 30 grain carotenoid abundance traits (Owens *et al.* 2014), 20 tocochromanol abundance traits (Lipka *et al.* 2013), and 22 field-measured agronomic traits (Hung *et al.* 2012).

### Integrating TWAS with GWAS improves power for identifying and prioritizing known genes

To assess the relative power of each method to detect known genes, we counted the number of known genes identified in the top 1% ranked genes (based on p-values) found by each method for each trait. This identification of known genes among the top 1% of hits for each method measures how often known genes appear in the tail of the distribution of detected genes and avoids direct comparisons of p-values between differently powered and structured tests that rely on continuous (TWAS) or discrete (GWAS) independent variables.

As shown in Tables 1, 2, S1, and S2, the combined test outperforms either the genotype-based or expression-based tests alone for both classes of traits, with 30 total detections of known genes among the top 1% of associations across tocochromanol and 75 detections of putative carotenoid related genes (Owens *et al.* 2014) when using the carotenoid traits. Using the tocochromanol and carotenoid lists from (Diepenbrock *et al.* 2017; Owens *et al.* 2014) genes are detected more often in each of the tocochromanol and carotenoid trait classes when using the combined method. However, the Fisher’s combined test of GWAS results with the multi-tissue TWAS results did not perform better. The detection rate was consistently higher for kernel-based TWAS over the multi-tissue TWAS, most likely because the tocochromanol and carotenoid traits are predominantly controlled by gene expression in the kernel.

**Table 1:** Summary of total and unique known gene detections in top 1% of results across tocochromanol traits by kernel TWAS with PEERS and PCs, multi-tissue TWAS with PCs, MLM GWAS, Fisher’s combined test of kernel TWAS with PEERS and PCs and MLM GWAS, and Fisher’s combined test of multi-tissue TWAS with PCs and MLM GWAS. There are 14 previously known tocochromanol genes in maize (Diepenbrock *et al.* 2017). On the left half of the table the number of detections exceeds the number of known genes because a gene is counted as detected each time it is in the top 1% of associations for the 20 tocochromanol component traits

Test	Detection of know genes in top 1% hits across tocochromanol traits
TWAS	14
multiTWAS	13
GWAS	21
FisherGWASTWAS	30
FisherGWASmultiTWAS	27

**Table 2:** Summary of total and unique putative carotenoid gene [28] detections in top 1% of results across carotenoid traits by kernel TWAS with PEERS and PCs, multi-tissue TWAS with PCs, MLM GWAS, Fisher’s combined test of kernel TWAS with PEERS and PCs and MLM GWAS, and Fisher’s combined test of multi-tissue TWAS with PCs and MLM GWAS. On the left half of the table the number of detections exceeds the number of known genes because a gene is counted as detected each time it is in the top 1% of associations for the 30 carotenoid component traits

Test	Detection of candidate genes in top 1% hits across carotenoid traits
TWAS	38
multiTWAS	32
GWAS	55
FisherGWASTWAS	75
FisherGWASmultiTWAS	58

**Figure 2:**
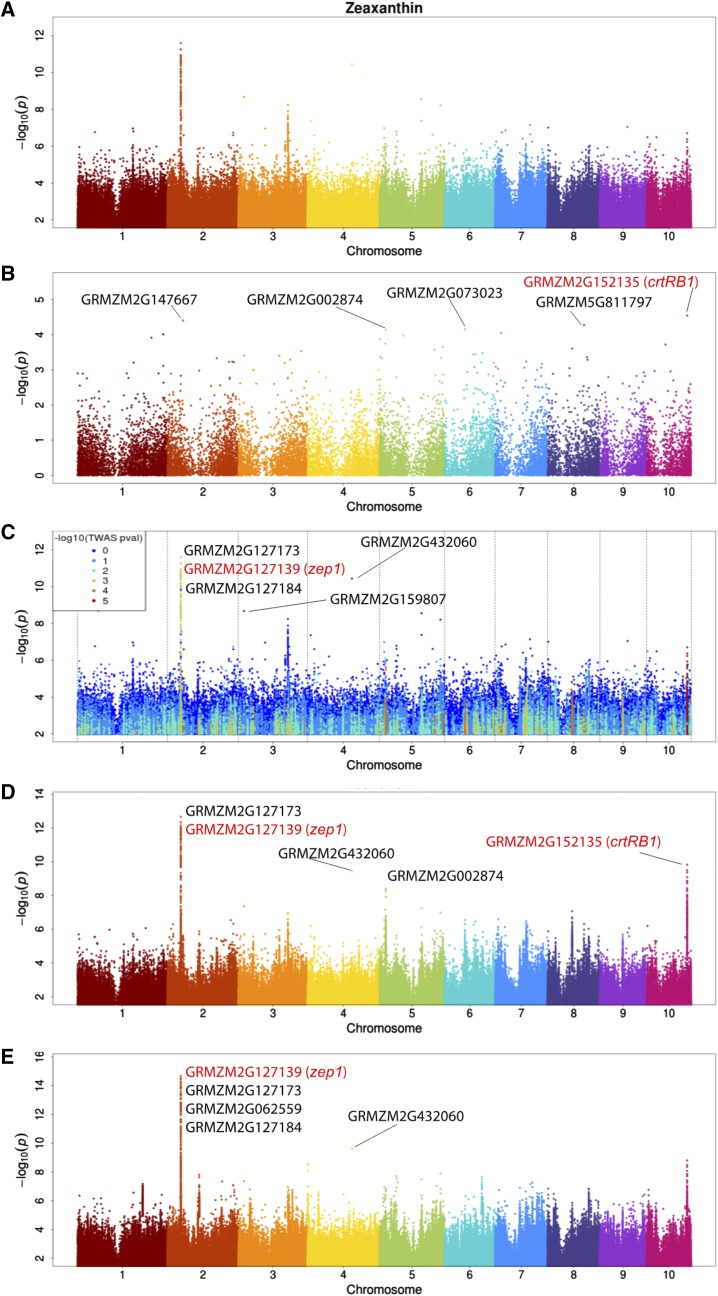
Manhattan plots of zeaxanthin abundance A) mixed linear model GWAS accounting for kinship, B) kernel TWAS with PEER and genetic MDS PC covariates, C) MLM colored by TWAS significance, and D) Fisher’s combined model of MLM and TWAS p-values using kernel expression. E) Fisher’s combined model of MLM and multi-tissue TWAS p-values. The top five most associated genes are labeled and previously identified genes by Owens *et al.* (2014) are highlighted in red.

We also compared the methods at the level of single traits. To determine how the combined method prioritizes genes that are not detected in the individual TWAS and GWAS methods and aggregates genes that are detected by only one method, we plotted the results across models for each individual trait. In Figure 2 we plotted the signals mapped for the zeaxanthin trait. Note that points representing SNPs from the MLM GWAS model in (c) and (a) are identically placed, but in (c) they are colored by TWAS significance. The top five genes detected by each method are labeled (a is not individually labeled because the points and top five genes are identical to those in plot c) and previously detected genes found by Owens *et al.* (2014) are highlighted in red. As shown by the TWAS results plotted in Figure 2B, the known expression-regulated gene *crtRB1* has expression which is most strongly correlated (r = 0.309, *P* = 2.84e-5) with zeaxanthin abundance in our TWAS model that includes genetic and expression-derived covariates (see methods). *crtRB1* is not among the top MLM GWAS-detected genes in our study, but the detection of *crtRB1* by kernel TWAS is consistent with previous results (Owens *et al.* 2014; Yan *et al.* 2010), highlighting this gene’s role as a principal determinant of grain carotenoids which acts through variable expression.

As is clear in Figure 2A, C another zeaxanthin-implicated gene, zeaxanthin epoxidase, *zep1*, is detected by GWAS in our study (Owens *et al.* 2014). *zep1* expression is correlated (r= 0.232, *P* = 0.0014) with zeaxanthin abundance, but it is not among the fifty most significantly associated genes in our TWAS results, and would not be prioritized by TWAS alone. However, within the peak covering *zep1* in Figure 2 A,C the markers most strongly associated with zeaxanthin from the MLM GWAS results prioritize a different gene first, GRMZM2G127123, which lacks a known function. The linkage-independent kernel TWAS results also show nearly equal support for both genes, providing evidence that GRMZM2G127123 (r= 0.218, *P* = 0.0025) and *zep1* (r= 0.232, *P* = 0.0014) both affect zeaxanthin abundance. Both Fisher’s combined models using the single-tissue and multi-tissue TWAS results also support the importance of both genes.

**Figure 3:**
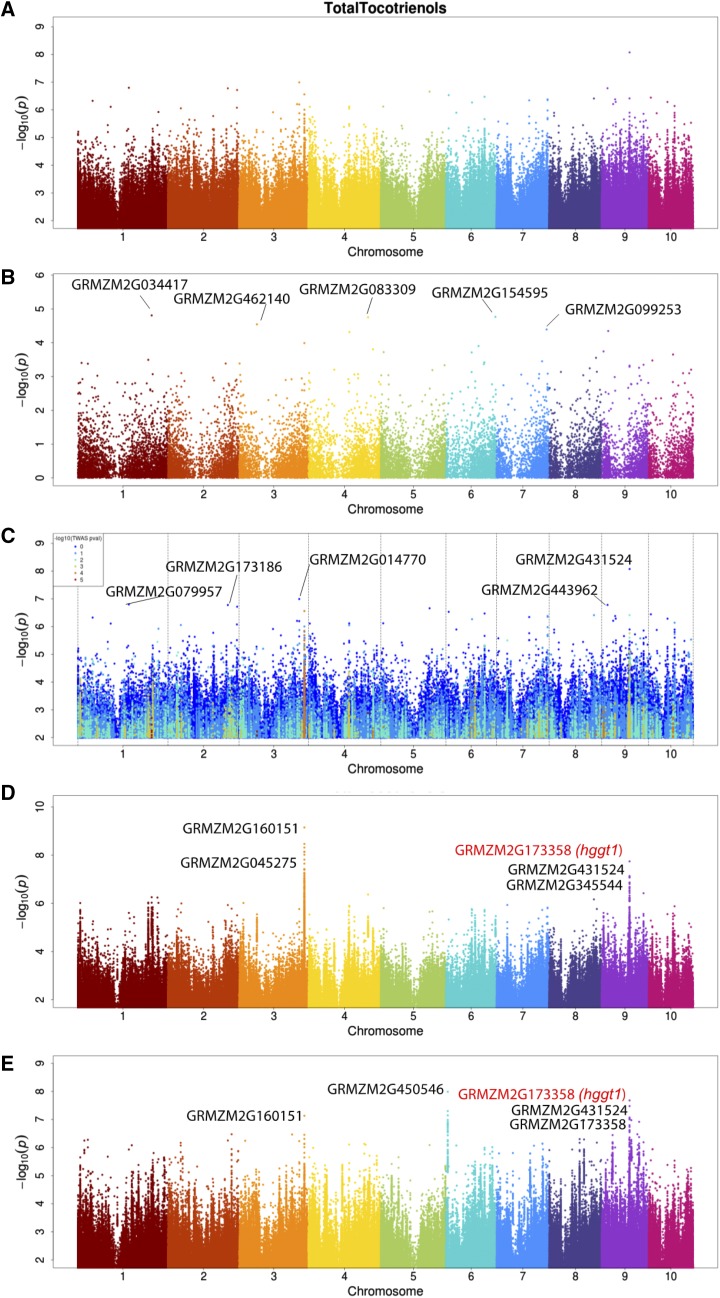
Manhattan plots of total tocotrienol abundance A) mixed linear model GWAS accounting for kinship, B) kernel TWAS with PEER and genetic MDS PC covariates, C) MLM colored by TWAS significance, and D) Fisher’s combined model of MLM and TWAS p-values using kernel expression. E) Fisher’s combined model of MLM and multi-tissue TWAS p-values. The top five most associated genes are labeled and previously identified genes Diepenbrock *et al.* (2017) are highlighted in red.

To test the capacity of the TWAS, GWAS, and combined methods to re-identify genes known to underlie QTL for another trait class, we examined the detected genes for the total tocotrienol trait measured by Lipka *et al.* (2013). In Figure 3 the most strongly associated variant identified by GWAS is on chromosome 9 nearest a gene of unknown function, GRMZM2G431524. However, as is illustrated in the MLM GWAS Manhattan plot in which points are colored by TWAS significance (c), the other points in the chromosome 9 peak are near other genes known to underlie QTL whose expression is variably associated with total tocotrienol abundance. These second and third most strongly associated genes based on proximity to the most significant markers identified by GWAS are GRMZM2G345544 (function unknown) and *hggt1*, which has been previously tied to total tocotrienol content (Lipka *et al.* 2013), and is essential for tocotrienol biosythesis. However, because *hggt1* expression is most strongly correlated with total tocotrienol measurements from among these first three genes in the chromosome 9 peak, the combined test using single tissue and multiple tissues of expression dat*a priori*tizes the known gene *hggt1* suggesting it is the functional gene in this region, consistent with previous evidence. This illustrates how the supplementary information from expression associations prioritizes likely causal genes that are not among the top hits of either individual expression or genotype-based methods.

### Variance component estimation from TWAS- and GWAS-detected genes

To further assess the capacity of each method to correctly identify genes affecting each trait, an independent variance partitioning approach (Gusev *et al.* 2014; Rodgers-Melnick *et al.* 2016; Speed *et al.* 2017) was also performed. Using variants in a 1 Mb window around the ten top ranked genes identified in the Goodman diversity panel (Flint-Garcia *et al.* 2005) by GWAS alone, TWAS alone, and the combined method, separate kinship matrices were calculated. These relationship matrices were fit as random effects in separate models of phenotypic variance explained for traits measured in the NAM population, which is largely independent of the Goodman diversity panel in which the various mapping strategies were performed. The additive genetic variance explained by the variants underlying each kinship matrix was calculated providing an estimate of heritability explained by the genes identified by each method.

**Figure 4:**
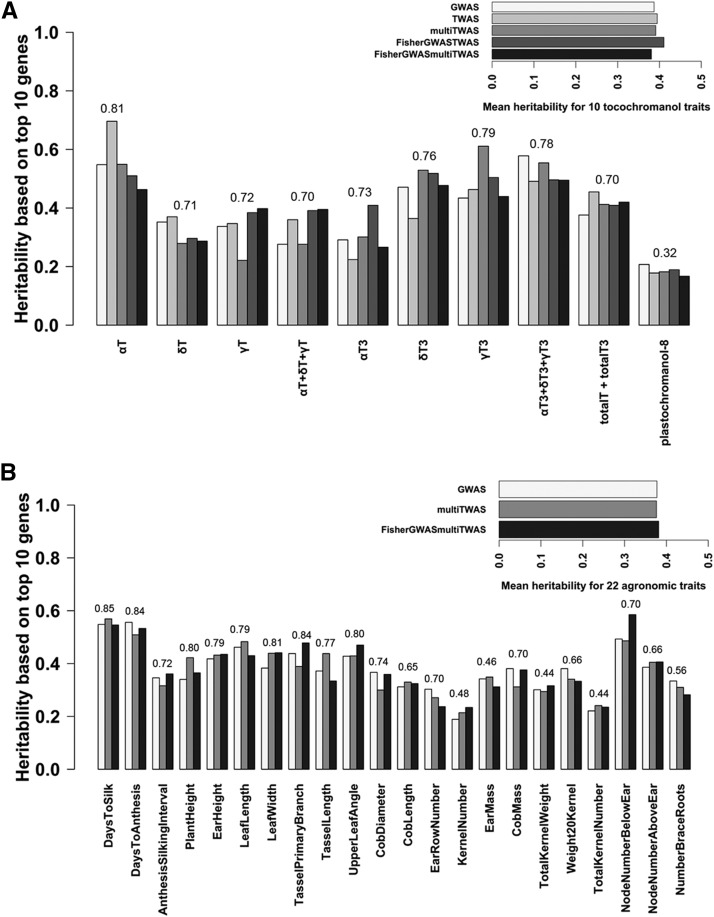
Variance partitioning of heritable variation using all NAM families for A) tocochromanol traits and B) agronomic traits. Vertical barplots represent the heritability estimated from kinship matrices made from the genetic regions adjacent to the top 10 ranked genes mapped by MLM GWAS, kernel-based TWAS, multi-tissue TWAS, the Fisher’s combined test of the MLM GWAS + kernel-based TWAS, and the Fisher’s combined test of the MLM GWAS + multi-tissue TWAS. Horizontal barplots compare model based on mean heritability across traits per trait class. Heritability explained by using all SNPs for each trait was put at the top of each grouped barplot.

Using variance partitioning across all NAM families, we found some advantage for including expression data in detecting likely functional regions of the genome (Figure 4). Among the tocochromanol kernel traits (Figure 4A), eight out of ten traits exist in which TWAS or the Fisher’s combined method is superior to GWAS alone (Figure 4A). Heritable variance explained on a per-trait basis by either the TWAS alone or the Fisher’s combined method showed about 25% improvement on average over the MLM GWAS, with notable advantage for alpha-tocotrienol (40%), gamma-tocotrienol (41%) and total tocopherol (43%). For more complex field-based agronomic traits, the multi-tissue TWAS or Fisher’s combined method also showed an advantage over GWAS alone in 16 out of 22 agronomic traits (Figure 3B). On average, the multi-tissue TWAS had 24% improvement over GWAS alone, while the FisherGWASmultiTWAS had notable advantage for kernel number (24%), leaf width (15%), and node number below ear (19%). Based on mean heritable variance across traits per trait class, the combined Fisher’s test explained the most heritability among the models; it showed 4–8% improvement for the tocochromanol kernel traits (Figure 4A inset). However, little improvement was observed for agronomic traits likely due to trait complexity (Figure 4B inset). Because previously known genes are more often re-identified in the top 1% of hits by combining GWAS and TWAS (Table 1), the variance explained by markers near detected genes also reflect this advantage on heritability with known oligogenic architecture.

**Figure 5:**
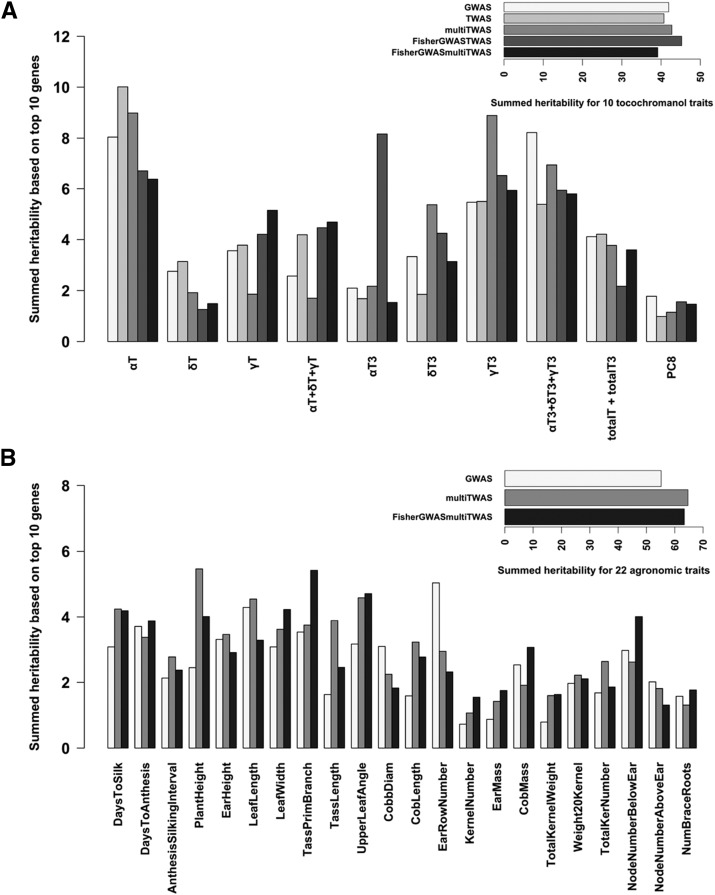
Family-based variance partitioning on individual NAM family. Heritability for each trait was estimated for each of 24 NAM families using kinship matrices made from the genetic regions adjacent to the top 10 ranked genes mapped by MLM GWAS, kernel-based TWAS, multi-tissue TWAS, the Fisher’s combined test of the MLM GWAS + kernel-based TWAS, and the Fisher’s combined test of the MLM GWAS + multi-tissue TWAS. There were a total of 24 independent tests for each trait-model combination. Heritability estimates were then added together (hereafter, summed heritability) for A) tocochromanol traits and B) agronomic traits. Horizontal barplots compare model based on total summed heritability across traits per trait class.

We further tested the heritability explained by the top ten ranked genes identified by each method using family-based variance partitioning (Figure 5). Heritable variance was decomposed for each NAM family, giving 24 independent tests of variance partitioning for each trait tallying a total of 3,840 independent tests (24 families * 5 models * 32 traits). To evaluate the best winning model for each trait, we took the sum of heritable variance across 24 NAM families (hereafter, summed heritability). Based on the same set of genes identified from each model, our results illustrate the differing levels of heritability among families for both tocochromanol (Fig. S1, Figure 5A) and agronomic traits (Fig. S2, and S3; Figure 5B). For α-tocotrienol, which is an oligogenic trait, the FisherGWASTWAS method explained the most heritability in 18 out of 24 NAM families (Figure S1a), giving a fourfold advantage on summed heritability over either GWAS or TWAS alone (Figure S1b; Figure 5A). The FisherGWASTWAS method captured the most summed heritability in 10 tocochromanol traits (Figure 5A inset), consistent with what we found in variance partitioning using all NAM families for tocochromanol traits (Figure 4A). On a per-trait basis, we note that the kernel-based TWAS or the FisherGWASTWAS was the winning method for eight out of 10 tocochromanol traits. We do see a similar pattern in 19 out of 22 field-based complex traits in which either the multi-tissue TWAS or FisherGWASmultiTissueTWAS explained the most heritability (Figure 5B). We see greater advantage of the FisherGWASmultiTissueTWAS over the GWAS MLM for tassel primary branch (54%), cob length (103%), kernel number (112%), ear mass (98%) and total kernel weight (106%) (Figure 5A). For the more complex traits such as plant height, the multi-tissue TWAS was the winning model, which explained about twofold higher heritability than the GWAS alone (Figure 5B, Fig. S3). We found that in 16 NAM families, the multi-tissue TWAS explained the most heritability among other models for plant height. Based on total summed heritability across 22 agronomic traits (Figure 5B inset), the FisherGWASmultiTissueTWAS and multi-tissue TWAS showed a 15% and 17% improvement in heritability explained over the GWAS MLM alone, respectively.

## Discussion

By far the majority of efforts to dissect the architecture of terminal phenotypes have relied on associations with genetic variants; this capacity to link genotype to phenotype has recently been accelerated by the plummeting cost of sequencing. The more recent advent of technologies which permit the quantification of endophenotypes like mRNA, metabolite, or protein abundance now enable mapping and trait dissection to be done between intermediate levels of biological organization. Assaying and associating these endophenotypes with traits of interest provides insight on biological mechanisms, serves as an independent source of evidence of associations, and facilitates prioritizing potentially causal variation while linking genes directly to traits in a way that potentially integrates the effects of multiple independent genetic variants. Here, we illustrated the utility of using a large RNA-seq resource in maize (Kremling *et al.* 2018) for transcriptome-wide association studies and integrating these results with associations based on genetic variation.

We find evidence supporting the inclusion of transcriptome-wide variation in addition to genetic variation in models seeking to associate traits to underlying and likely causal genes in diverse maize lines, especially when the goal is to infer function of genes underlying oligogenic traits. Across tocochromanol trait classes, the inclusion of TWAS results enables more frequent detection of known causal genes and helps to prioritize novel candidate genes in the profiled panel. Crucially, transcriptional variation alone does not improve over genotype-based associations, but it is in combination with genotypic information that the power of gene detection is increased.

As we demonstrate here, TWAS in combination with GWAS enhances the capacity to prioritize candidate genes over the use of GWAS alone. Given that more than half of detections are supported by TWAS (Table 1), our results also reveal much of the functional variation for these traits to be regulatory. While not all previously identified genes are detected by TWAS, this is likely a combination of insufficient power compared to the previous association studies in the NAM population with >16x as many observations (Diepenbrock *et al.* 2017), the sampling of a single time point per tissue, and the fact that not all functional variation is regulatory. Despite these limitations, TWAS adds value to GWAS mapping alone and increases the power to re-detect known genes. Our finding that TWAS alone is a valid method for finding true gene-trait associations is consistent with the recent findings of Lin *et al.* (2017) and colleagues despite the difference between the eRD-GWAS and TWAS models. However, our results differ in that we demonstrate that a combined test integrating TWAS and GWAS yields a more powerful test than either method individually when it comes to re-identifying known genes underlying oligogenic traits (Diepenbrock *et al.* 2017).

We also note that our efforts to validate our TWAS and GWAS detections differ from Lin *et al.* (2017). In contrast to comparing the overlap of the detections by GWAS and TWAS in the same study, we compared our detections to previously known genes found in a largely independent set of germplasm, namely the NAM population (Yu *et al.* 2008), which was used to find tocochromanol associations (Diepenbrock *et al.* 2017). Also, in contrast to the previously published study, we did not perform our cross-validation analysis in the same set of germplasm in which discovery was conducted by GWAS and TWAS to assess accuracy. Using variance partitioning in the largely independent NAM population, we found similar levels of variance explained by the genes detected by each method in the Goodman diversity panel (Flint-Garcia *et al.* 2005), illustrating that even when the identified genes are tested in an outside population, the detections of the transcriptome-only and combined methods are found to be valid and explain similar amounts of variance to the genotype-based methods (Figures 4, 5). This is roughly consistent with the cross-validation results comparing SNP_BayesB and eRD-GWAS presented in Table S4 by Lin and colleagues (Lin *et al.* 2017). However, the previously published results show an advantage for eRD-GWAS for only one of fourteen traits, while on the basis of variance partitioning for kernel traits we find an advantage for the kernel-based TWAS or the Fisher’s combined model for nine of the ten kernel-based traits for which measurements in NAM exist.

In further contrast to the previously published work (Lin *et al.* 2017), none of the SNPs used in our GWAS or variance partitioning methods were derived from RNA-seq data, allowing for less bias toward expressed genes and giving the genotype-based tests more independence from the expression-based tests. In the previous work, more than 0.9M of the 1.2 M genetic variants were derived from the alignment of RNA-seq reads (Leiboff *et al.* 2015; Lin *et al.* 2017), potentially confounding the ability to make associations by GWAS with the presence of an expressed gene, and thus limiting the power of the genotype-based GWAS to make associations which are independent of expression.

It is striking that even in diverse maize lines where linkage decays quickly (Wallace *et al.* 2014) and thus the power to resolve mapping peaks to individual genes is high, TWAS provides a valuable supplement to genetic mapping alone. This benefit of TWAS would be compounded in species or populations in which resolution is limited. Additionally, by imputing expression values based on local/*cis* haplotype, as has been successfully shown in humans (Pasaniuc and Price 2017), the utility of TWAS could potentially be extended further in maize. Imputing expression to a larger panel would permit the exploitation of previously measured phenotypes across a much larger set of individuals which have not been expression profiled. By imputing only the local/*cis* genetic component of expression, and implicitly averaging over *trans* and environmental effects, the capacity to attribute field phenotypes to the genetic component of expression would likely be further improved.

The lack of improvement in re-detecting known tocochromanol traits by the multi-tissue TWAS models alone or as part of the Fisher’s combined tests is notable, but unsurprising for these genetically simple and very tissue specific traits. This lack of improvement indicates that kernel-based expression alone is most predictive of the kernel-based metabolites and accuracy is not improved by the incorporation of all other tissues. Rather than comparing the inclusion of all tissues *vs.* kernels only, in the future a variable (tissue) selection TWAS approach should be used in which can remove uninformative terms from the model rather than including them but giving them a very small coefficient. It is also plausible that for more genetically complex traits which are also affected by expression across tissues, the multi-tissue TWAS results are more likely to be informative.

A further cause of the limited improvement for the kernel TWAS or Fisher’s combined test seen in the variance partitioning results is likely because GWAS identifies genomic regions which, when expanded to a 1 Mb window, could cover the functional variants. Furthermore, while the correct functional gene may not be prioritized by GWAS, if the trait is affected by genetic regulation rather than coding sequence change, the sites near the GWAS hit may in fact be more functional than those near the mechanistically significant gene itself even if they are misattributed to the incorrect proximal gene. Using a large independent diverse panel with very low LD to assess the heritability explained by the SNPs identified by each method may also provide a better estimate as the functional variants are not as easily tagged over long distances.

While the utility of expression endophenotypes in dissecting traits has been demonstrated here, it should be noted that associations made between endophenotypes and terminal phenotypes are inherently more susceptible to environmental effects than genotype-based associations. This susceptibility to environmental effects likely allows us to associate only the environmentally independent heritable fraction of expression with phenotype in our study, especially because expression data were collected from separate plants than those for which terminal phenotypes were measured. Given that in endophenotype-based association studies, like TWAS, environmental variation separately impacts and increases error in both the independent and dependent variables, methods like TWAS alone may plausibly be expected to perform more poorly than genetics-based associations. However, this shortcoming is partially compensated for by the more direct link between endophenotype and terminal phenotype and the potential discovery of mechanism. The collection of expression data from the same plants and conditions in which the phenotypes are collected would likely benefit the dissection of genotype by environment interactions by highlighting the impact of variation in expression for a specific gene within an environment, but cannot be examined here as terminal phenotypes and expression values were calculated from separate environments and years.

## References

[bib1] Albert F. W., and Kruglyak L., 2015 The role of regulatory variation in complex traits and disease. Nat. Rev. Genet. 16: 197–212. 10.1038/nrg3891 25707927

[bib2] Battle A., Khan Z., Wang S. H., Mitrano A., Ford M. J., 2015 Genomic variation. impact of regulatory variation from RNA to protein. Science 347: 664–667. 10.1126/science.1260793 25657249 PMC4507520

[bib3] Bradbury P. J., Zhang Z., Kroon D. E., Casstevens T. M., Ramdoss Y., 2007 TASSEL: Software for association mapping of complex traits in diverse samples. Bioinformatics 23: 2633–2635. 10.1093/bioinformatics/btm308 17586829

[bib4] Bukowski R., Guo X., Lu Y., Zou C., He B., 2018 Construction of the third generation zea mays haplotype map. Gigascience 7: 1–12. 10.1093/gigascience/gix134 PMC5890452 29300887

[bib5] Carroll S. B., 2008 Evo-devo and an expanding evolutionary synthesis: A genetic theory of morphological evolution. Cell 134: 25–36. 10.1016/j.cell.2008.06.030 18614008

[bib6] Chick J. M., Munger S. C., Simecek P., Huttlin E. L., Choi K., 2016 Defining the consequences of genetic variation on a proteome-wide scale. Nature 534: 500–505. 10.1038/nature18270 27309819 PMC5292866

[bib7] Dewey M., 2017 Metap: Meta-analysis of significance values r-package.

[bib8] Dickson S. P., Wang K., Hakonarson H., and Goldstein D. B., 2010 Rare variants create synthetic genome-wide associations. PLoS Biol. 8: e1000294 10.1371/journal.pbio.1000294 20126254 PMC2811148

[bib9] Diepenbrock C. H., Kandianis C. B., Lipka A. E., Magallanes-Lundback M., Vaillancourt B., 2017 Novel loci underlie natural variation in vitamin E levels in maize grain. Plant Cell 29: 2374–2392. 10.1105/tpc.17.00475 28970338 PMC5774569

[bib10] Dubreuil P., and Charcosset A., 1998 Genetic diversity within and among maize populations: A comparison between isozyme and nuclear RFLP loci. TAG 96: 577–587. 10.1007/s001220050776

[bib11] Flint-Garcia S. A., Thuillet A. C., Yu J., Pressoir G., Romero S. M., 2005 Maize association population: A high-resolution platform for quantitative trait locus dissection. Plant J. 44: 1054–1064. 10.1111/j.1365-313X.2005.02591.x 16359397

[bib12] Fu J., Cheng Y., Linghu J., Yang X., Kang L., 2013 RNA sequencing reveals the complex regulatory network in the maize kernel. Nat. Commun. 4: 2832 10.1038/ncomms3832 24343161

[bib13] Gibson G., 2012 Rare and common variants: Twenty arguments. Nat. Rev. Genet. 13: 135–145. 10.1038/nrg3118 22251874 PMC4408201

[bib14] GTEx Consortium, 2015 Human genomics. the genotype-tissue expression (GTEx) pilot analysis: Multitissue gene regulation in humans. Science 348: 648–660. 10.1126/science.1262110 25954001 PMC4547484

[bib15] Gusev A., Lee S. H., Trynka G., Finucane H., Vilhjalmsson B. J., 2014 Partitioning heritability of regulatory and cell-type-specific variants across 11 common diseases. Am. J. Hum. Genet. 95: 535–552. 10.1016/j.ajhg.2014.10.004 25439723 PMC4225595

[bib16] Gusev A., Ko A., Shi H., Bhatia G., Chung W., 2016 Integrative approaches for large-scale transcriptome-wide association studies. Nat. Genet. 48: 245–252. 10.1038/ng.3506 26854917 PMC4767558

[bib18] Harjes C. E., Rocheford T. R., Bai L., Brutnell T. P., Kandianis C. B., 2008 Natural genetic variation in lycopene epsilon cyclase tapped for maize biofortification. Science 319: 330–333. 10.1126/science.1150255 18202289 PMC2933658

[bib19] Henn B. M., Botigue L. R., Bustamante C. D., Clark A. G., and Gravel S., 2015 Estimating the mutation load in human genomes. Nat. Rev. Genet. 16: 333–343. 10.1038/nrg3931 25963372 PMC4959039

[bib20] Hirsch C. N., Foerster J. M., Johnson J. M., Sekhon R. S., Muttoni G., 2014 Insights into the maize pan-genome and pan-transcriptome. Plant Cell 26: 121–135. 10.1105/tpc.113.119982 24488960 PMC3963563

[bib21] Hufford M. B., Xu X., van Heerwaarden J., Pyhajarvi T., Chia J. M., 2012 Comparative population genomics of maize domestication and improvement. Nat. Genet. 44: 808–811. 10.1038/ng.2309 22660546 PMC5531767

[bib22] Hung H. Y., Browne C., Guill K., Coles N., Eller M., 2012 The relationship between parental genetic or phenotypic divergence and progeny variation in the maize nested association mapping population. Heredity 108: 490–499. 10.1038/hdy.2011.103 22027895 PMC3330692

[bib23] Juntawong P., Girke T., Bazin J., and Bailey-Serres J., 2014 Translational dynamics revealed by genome-wide profiling of ribosome footprints in arabidopsis. Proc. Natl. Acad. Sci. USA 111: E203–E212. 10.1073/pnas.1317811111 24367078 PMC3890782

[bib24] Kremling K. A., Chen S., Su M., Lepak N., Romay C. M., 2018 Dysregulation of expression correlates with rare allele burden and fitness loss in maize. Nature 555: 520–523. 10.1038/nature25966 29539638

[bib25] Law J. A., and Jacobsen S. E., 2010 Establishing, maintaining and modifying DNA methylation patterns in plants and animals. Nat. Rev. Genet. 11: 204–220. 10.1038/nrg2719 20142834 PMC3034103

[bib26] Leiboff S., Li X., Hu H. C., Todt N., Yang J., 2015 Genetic control of morphometric diversity in the maize shoot apical meristem. Nat. Commun. 6: 8974 10.1038/ncomms9974 26584889 PMC4673881

[bib27] Lin H. Y., Liu Q., Li X., Yang J., Liu S., 2017 Substantial contribution of genetic variation in the expression of transcription factors to phenotypic variation revealed by eRD-GWAS. Genome Biol. 18: 192-017-1328-6. 10.1186/s13059-017-1328-6 PMC5645915 29041960

[bib28] Lipka A. E., Gore M. A., Magallanes-Lundback M., Mesberg A., Lin H., 2013 Genome-wide association study and pathway-level analysis of tocochromanol levels in maize grain. G3 (Bethesda) 3: 1287–1299. 10.1534/g3.113.006148 23733887 PMC3737168

[bib29] Lippert C., Listgarten J., Liu Y., Kadie C. M., Davidson R. I., 2011 FaST linear mixed models for genome-wide association studies. Nat. Methods 8: 833–835. 10.1038/nmeth.1681 21892150

[bib30] Mancuso N., Shi H., Goddard P., Kichaev G., Gusev A., 2017 Integrating Gene Expression with Summary Association Statistics to Identify Genes Associated with 30 complex traits. Am. J. Hum. Genet. 100: 473–487. 10.1016/j.ajhg.2017.01.031 28238358 PMC5339290

[bib31] Mayr E., 1970 *Populations*, *species*, *and evolution: An abridgment of animal species and evolution*, Harvard University Press, Cambridge, MA.

[bib32] Owens B. F., Lipka A. E., Magallanes-Lundback M., Tiede T., Diepenbrock C. H., 2014 A foundation for provitamin A biofortification of maize: Genome-wide association and genomic prediction models of carotenoid levels. Genetics 198: 1699–1716. 10.1534/genetics.114.169979 25258377 PMC4256781

[bib33] Pasaniuc B., and Price A. L., 2017 Dissecting the genetics of complex traits using summary association statistics. Nat. Rev. Genet. 18: 117–127. 10.1038/nrg.2016.142 27840428 PMC5449190

[bib34] Riedelsheimer C., Czedik-Eysenberg A., Grieder C., Lisec J., Technow F., 2012 Genomic and metabolic prediction of complex heterotic traits in hybrid maize. Nat. Genet. 44: 217–220. 10.1038/ng.1033 22246502

[bib35] Rodgers-Melnick E., Vera D. L., Bass H. W., and Buckler E. S., 2016 Open chromatin reveals the functional maize genome. Proc. Natl. Acad. Sci. USA 113: E3177–E3184. 10.1073/pnas.1525244113 27185945 PMC4896728

[bib36] Shabalin A. A., 2012 Matrix eQTL: Ultra fast eQTL analysis via large matrix operations. Bioinformatics 28: 1353–1358. 10.1093/bioinformatics/bts163 22492648 PMC3348564

[bib37] Speed D., Cai N., Johnson M. R., Nejentsev S., and Balding D. J.; UCLEB Consortium, 2017 Reevaluation of SNP heritability in complex human traits. Nat. Genet. 49: 986–992. 10.1038/ng.3865 28530675 PMC5493198

[bib38] Tishkoff S. A., Reed F. A., Ranciaro A., Voight B. F., Babbitt C. C., 2007 Convergent adaptation of human lactase persistence in africa and europe. Nat. Genet. 39: 31–40. 10.1038/ng1946 17159977 PMC2672153

[bib40] Wallace J. G., Bradbury P. J., Zhang N., Gibon Y., Stitt M., 2014 Association mapping across numerous traits reveals patterns of functional variation in maize. PLoS Genet. 10: e1004845 10.1371/journal.pgen.1004845 25474422 PMC4256217

[bib41] Welter D., MacArthur J., Morales J., Burdett T., Hall P., 2014 The NHGRI GWAS catalog, a curated resource of SNP-trait associations. Nucleic Acids Res. 42: D1001–D1006. 10.1093/nar/gkt1229 24316577 PMC3965119

[bib42] Wisser R. J., Kolkman J. M., Patzoldt M. E., Holland J. B., Yu J., 2011 Multivariate analysis of maize disease resistances suggests a pleiotropic genetic basis and implicates a GST gene. Proc. Natl. Acad. Sci. USA 108: 7339–7344. 10.1073/pnas.1011739108 21490302 PMC3088610

[bib43] Yan J., Kandianis C. B., Harjes C. E., Bai L., Kim E. H., 2010 Rare genetic variation at *Zea mays* crtRB1 increases beta-carotene in maize grain. Nat. Genet. 42: 322–327. 10.1038/ng.551 20305664

[bib44] Yu J., Holland J. B., McMullen M. D., and Buckler E. S., 2008 Genetic design and statistical power of nested association mapping in maize. Genetics 178: 539–551. 10.1534/genetics.107.074245 18202393 PMC2206100

